# Therapeutic role of granulocyte-colony stimulating factor and aspirin in unexplained recurrent pregnancy loss: a retrospective study

**DOI:** 10.3389/fmed.2026.1857890

**Published:** 2026-10-07

**Authors:** Xia Huang, Qiongxiu Luo, Yuehua Luo, Yue Xiao

**Affiliations:** 1 Ji’an City Central People’s Hospital South Hospital, Ji’an, Jiangxi, China; 2 Ji’an Maternal and Child Health Hospital, Ji’an, Jiangxi, China

**Keywords:** granulocyte-colony stimulating factor (rhG-CSF), low-dose aspirin, miscarriage, retrospective observational study, stillbirth

## Abstract

**Background:**

Recurrent pregnancy loss (RPL), the loss of two or more clinically confirmed pregnancies, is observed in approximately 1%–5% of couples trying to conceive. Immune dysfunction is increasingly recognized as a contributing factor. This study evaluated pregnancy outcomes among women with unexplained RPL treated with combined recombinant human granulocyte-colony stimulating factor (rhG-CSF) and low-dose aspirin, while acknowledging that the evidence base for rhG-CSF in unexplained RPL remains uncertain.

**Methods:**

In this retrospective study, 300 women aged 18–45 years with at least two consecutive unexplained pregnancy losses were treated with subcutaneous rhG-CSF and low-dose aspirin. Eligible women had documented ovulation, no major uterine abnormality, and no male-factor infertility. Source records were reviewed for mode of conception; all 300 index pregnancies were documented as spontaneous conceptions, and no pregnancies achieved by assisted reproductive technology (ART) were included. rhG-CSF was initiated on the sixth day after ovulation and continued until menstruation or completion of 9 gestational weeks; aspirin 75 mg/day was initiated at the same post-ovulatory time point and continued until 36 gestational weeks unless pregnancy loss or a safety indication required discontinuation. Clinical pregnancy, live birth, miscarriage, stillbirth, neonatal outcomes, and adverse events were assessed. Maternal age was treated as a clinically relevant covariate. Analyses were conducted using IBM SPSS version 27.

**Results:**

Live birth at or after 20 weeks of gestation occurred in 211/300 women (70.3%) according to the institutional obstetric registry definition used in the source records. All index pregnancies were verified as spontaneous conceptions, with no ART pregnancies included. Clinical pregnancy, defined as an intrauterine gestational sac with fetal cardiac activity at 6–7 gestational weeks, was observed in 99.3% of participants. In the age-adjusted model, a higher pretreatment systolic/diastolic ratio remained associated with lower odds of live birth (adjusted OR = 0.34, 95% CI 0.22–0.52, *p* < 0.001), while increasing maternal age was also associated with lower live-birth odds (adjusted OR per year = 0.94, 95% CI 0.89–0.99, *p* = 0.031). The miscarriage rate was 29.7%, median neonatal birth weight was 3,012 g, and congenital anomalies were documented in 12.6% of recorded neonatal outcomes.

**Conclusion:**

In this retrospective cohort, combined rhG-CSF and aspirin therapy was associated with improved uterine Doppler indices and a 70.3% live-birth rate among women with unexplained RPL. Because the study lacked an untreated control group and rhG-CSF use for unexplained RPL remains off-label with conflicting trial evidence, these findings should be interpreted cautiously and confirmed in adequately powered controlled studies.

## 1 Introduction

Recurrent pregnancy loss (RPL) is defined as the loss of two or more clinically confirmed pregnancies, which occurs in roughly 1%–5% of couples trying to conceive (1, 2). RPL is associated with a severe emotional burden. Therefore, recognition and diagnosis after two losses rather than three could accelerate the evaluation process. The etiology of RPL is variable, including genetic abnormalities such as embryonic aneuploidy and parental translocations, uterine structural defects, endocrine disorders, chronic infections, and autoimmune conditions like antiphospholipid syndrome (3). Notably, antiphospholipid antibodies are found in approximately 15%–20% of women with unexplained RPL (2). Despite comprehensive workup, half of RPL cases have no clear etiology, underscoring the consideration of other mechanisms (4).

Immune dysfunction is increasingly implicated in unexplained RPL. Successful pregnancy requires maternal–fetal immune tolerance since failure of this balance can lead to rejection of the semi-allogeneic embryo (5). Indeed, many women with RPL show evidence of immune dysregulation, such as abnormal activity of T-cell or natural killer (NK) cells, skewed Th1/Th2 cytokine ratios, or autoantibodies (2, 6). Approximately 20% of RPL cases are accompanied by detectable autoimmune features (2). Such observations have led to the hypothesis that occult immune disturbances underlie many idiopathic miscarriages.

Recently, novel immunotherapies such as recombinant human granulocyte-colony stimulating factor (rhG-CSF) have been explored. Although G-CSF is classically used to stimulate neutrophil production, experimental and reproductive-medicine studies suggest possible effects on trophoblast proliferation, trophoblast invasion, endometrial receptivity, and maternal-fetal immune tolerance (7, 8). However, clinical evidence in unexplained RPL remains conflicting. Scarpellini and Sbracia reported improved live birth after G-CSF in a small randomized trial, whereas the larger randomized trial by Eapen et al. did not show improvement in pregnancy outcomes (9, 10). A 2023 systematic review reported improvement in conception rate but not a statistically significant improvement in live birth, and the 2022 ESHRE guideline states that there is no evidence to recommend G-CSF for women with unexplained RPL (11, 12).

Low-dose aspirin irreversibly inhibits platelet aggregation and reduces pro-inflammatory prostaglandins through its anti-inflammatory and antithrombotic effects, which may improve uteroplacental blood flow. In practice, aspirin is standard care for RPL associated with antiphospholipid antibodies and is frequently used empirically in other RPL patients (4, 13). Aspirin may prevent microthrombosis at the implantation site and enhance trophoblast invasion with subsequent improved placental circulation.

Combining rhG-CSF with aspirin may leverage complementary immunologic and vascular mechanisms. G-CSF may influence endometrial receptivity and maternal-fetal immune regulation, while low-dose aspirin may improve uteroplacental perfusion through antiplatelet and anti-inflammatory effects. A recent retrospective study reported higher live-birth rates and improved uterine artery Doppler indices among women receiving rhG-CSF plus low-dose aspirin compared with untreated controls (14). Nevertheless, use of rhG-CSF for unexplained RPL remains off-label. Regulatory labeling for filgrastim products covers hematologic, oncologic, stem-cell mobilization, and radiation-related indications rather than RPL (15). Pregnancy safety data from labeling and observational neutropenia registries have not established an association with major birth defects, miscarriage, or adverse maternal/fetal outcomes, but these data are not specific to unexplained RPL and do not establish efficacy (15–17).

Therefore, this study aims to assess the effect of adding rhG-CSF to aspirin on endometrial receptivity, clinical pregnancy and live-birth outcomes in women with RPL.

## 2 Methods

### 2.1 Study design and setting

This retrospective analysis evaluated clinical pregnancy outcomes, uterine receptivity, and neonatal outcomes among women with unexplained RPL who received combined rhG-CSF and aspirin at Ji’an City Central People’s Hospital South Hospital and Ji’an Maternal and Child Health Hospital between 1 August 2024 and 1 July 2025. The institutional review board approved the study (approval number: 2025-L00201) and waived informed consent because of the retrospective design. Patient data were coded and de-identified before analysis. The study adhered to the Declaration of Helsinki and applicable data-protection regulations. Because rhG-CSF is not guideline-recommended for routine unexplained RPL care, its use in this cohort should be interpreted as off-label treatment delivered in local clinical practice rather than established standard therapy.

### 2.2 Participants

The study enrolled women aged 18–45 years with a documented history of two or more consecutive unexplained pregnancy losses, with at least two losses confirmed by ultrasound or histology. The 18–45-year range was selected to reflect the reproductive-age population treated in routine RPL practice; because maternal age older than 35 years is a recognized risk factor for pregnancy loss, maternal age was prespecified as a potential confounder in the statistical analysis. Eligible women had normal menstrual function, ultrasound-confirmed ovulation, endometrial thickness not exceeding 7.0 mm at the anticipated time of conception, and partner semen analysis showing no abnormality consistent with male-factor infertility. Mode of conception was verified by review of the source obstetric and fertility records. Pregnancies achieved through *in vitro* fertilization, intracytoplasmic sperm injection, intrauterine insemination, ovulation-induction cycles documented as ART, or other assisted reproductive technologies were excluded from the primary analysis. All 300 index pregnancies included in the analysis were documented as spontaneous conceptions.

Women with structural uterine abnormalities that could impair endometrial development, such as congenital malformations, intrauterine adhesions, or other anatomical variations, were excluded from the analysis. Moreover, patients with unexplained vaginal bleeding, a preexisting diagnosis of infertility from causes other than recurrent pregnancy loss and allergy to study medications were not eligible for this study.

### 2.3 Treatment protocol

Participants received combined rhG-CSF and low-dose aspirin. rhG-CSF was delivered by subcutaneous injection using single-use prefilled syringes beginning on the sixth day after ovulation and continuing until either onset of menstruation or completion of the first nine gestational weeks. The rhG-CSF dose was standardized at 100,000 IU/kg/day. Aspirin was initiated concurrently on the sixth day after ovulation at 75 mg orally once daily and was continued until 36 gestational weeks unless spontaneous abortion, ectopic pregnancy, premature labor, clinically significant bleeding, hypersensitivity, or another safety indication required earlier discontinuation (Figure 1).

**Figure 1:**
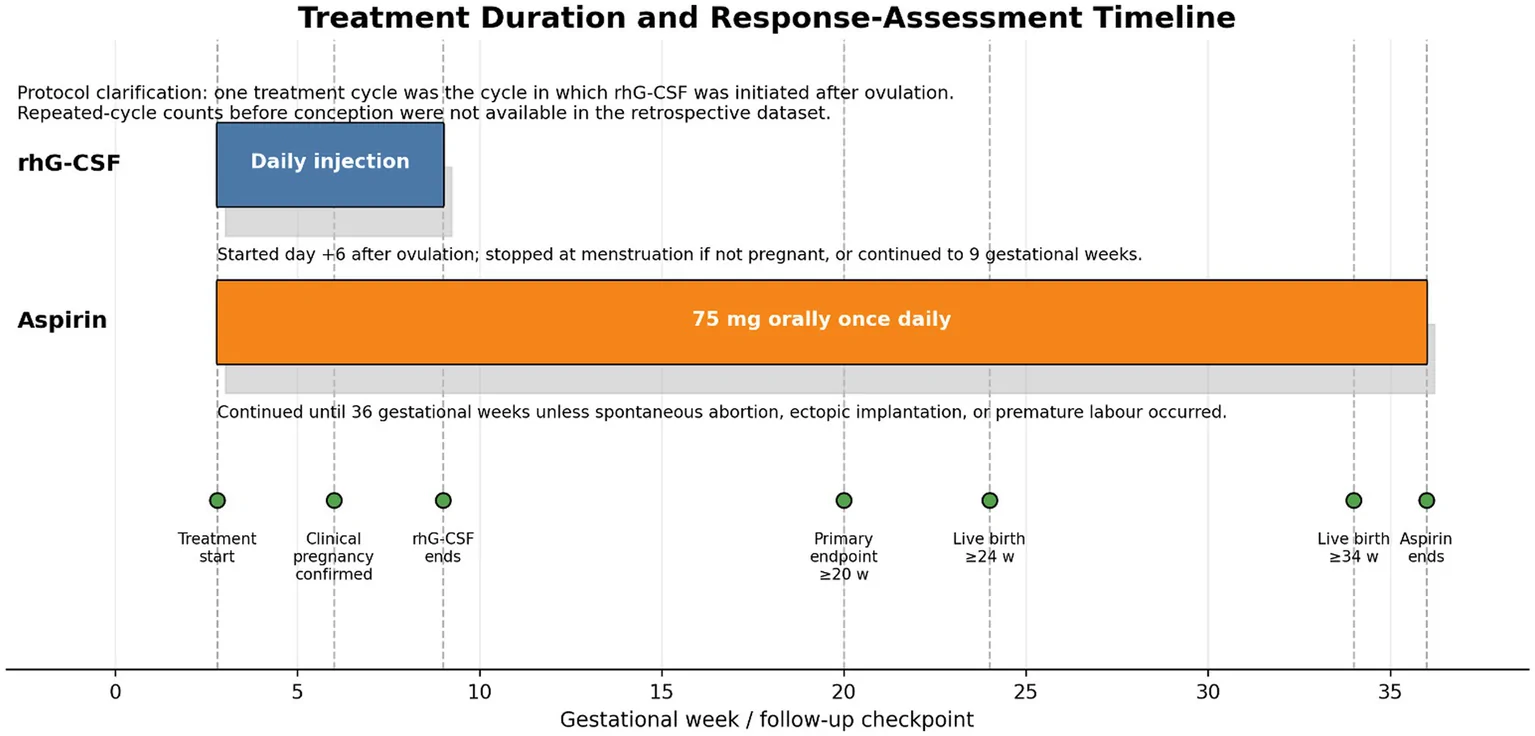
Treatment duration and response-assessment timeline. rhG-CSF was administered by daily subcutaneous injection from day 6 after ovulation until menstruation if pregnancy did not occur or until 9 gestational weeks if pregnancy continued. Aspirin (75 mg orally once daily) was administered from day 6 after ovulation until 36 gestational weeks unless pregnancy loss or a safety indication required discontinuation. Green markers indicate the treatment start and key outcome-assessment time points. The timeline represents one treatment cycle.

### 2.4 Data collection and outcome assessment

Demographic data, including age, ethnicity/nationality, body mass index (BMI), smoking status, alcohol use, coexisting comorbidities, and previous obstetric history, were retrospectively collected from hospital registration records and clinical charts. Race/ethnicity was not genetically inferred; it was recorded as an administrative hospital variable based on routine registration data. After data-dictionary review, the categories were revised from the template-derived labels of White/Black/Asian to the source categories used for this cohort: Han Chinese, Chinese ethnic minority, and other/not documented. Doppler ultrasound was used during follow-up to assess endometrial receptivity through endometrial blood-flow resistive index, pulsatility index, and systolic-to-diastolic ratio. The primary endpoint was live birth at or after 20+0 weeks of gestation, defined as delivery of an infant showing signs of life at or beyond the institutional registry cutoff that separated miscarriage from delivery outcomes. Secondary outcomes included live birth at or after 24 and 34 weeks, clinical pregnancy assessed by transvaginal ultrasound at 6+0 to 7+0 weeks and defined as an intrauterine gestational sac with fetal cardiac activity, stillbirth, preterm birth, miscarriage during follow-up, neonatal birth weight, congenital anomalies, and adverse events related to therapy.

### 2.5 Statistical analysis

Statistical analyses were performed using IBM SPSS Statistics version 27. Baseline continuous variables were summarized as medians and interquartile ranges (IQRs), and categorical variables as numbers and percentages. Doppler indices before and after treatment were compared using the paired Wilcoxon signed-rank test; two-sided *p*-values < 0.05 were considered statistically significant. For the primary endpoint (live birth ≥20 weeks), we fitted multivariable logistic-regression models reporting adjusted odds ratios (ORs) with 95% confidence intervals (CIs). Maternal age was prespecified as a clinically relevant confounder and was entered into the primary model as a continuous variable. A sensitivity analysis categorized maternal age as >35 versus ≤35 years. The pretreatment systolic-to-diastolic ratio was retained as the main Doppler predictor. Secondary and safety outcomes were summarized descriptively. Pairwise deletion was applied for missing data.

## 3 Results

### 3.1 Baseline characteristics and demographic data

We enrolled 300 women with a median age of 32 years (22–35) and median BMI of 22.9 kg/m^2^ (20–25.1). After review of the source data dictionary, the demographic variable was reported as ethnicity/nationality rather than Western race categories: 252 participants (84.0%) were recorded as Han Chinese, 29 (9.7%) as Chinese ethnic minority, and 19 (6.3%) as other/not documented. These categories reflect routine hospital registration data in the local Jiangxi recruitment setting and are not intended to represent genetic ancestry. Mode of conception was verified from source records: 300/300 pregnancies (100.0%) were spontaneous conceptions, and no ART pregnancies were identified. Overall, 56.1% of participants were nonsmokers, 23.7% smoked <10 cigarettes/day, 11.5% smoked 10-19 cigarettes/day, and 8.7% smoked ≥20 cigarettes/day. Alcohol use was recorded in the clinical chart; 46.0% reported no current alcohol use, 33.0% reported ≤3 units/day, 12.0% reported 4–7 units/day, and 9.1% reported >7 units/day. Regarding prior obstetric history, 57.7% had no previous live birth, 20.1% had four or more previous miscarriages, and the median number of previous losses was 3 (2–4). Comorbid gynecologic factors included polycystic ovaries in 58 women (20.3%), uterine fibroids in 36 (12.2%), and previous large-loop excision of the transformation zone in 28 (9.4%) (Figure 2; Table 1).

**Figure 2:**
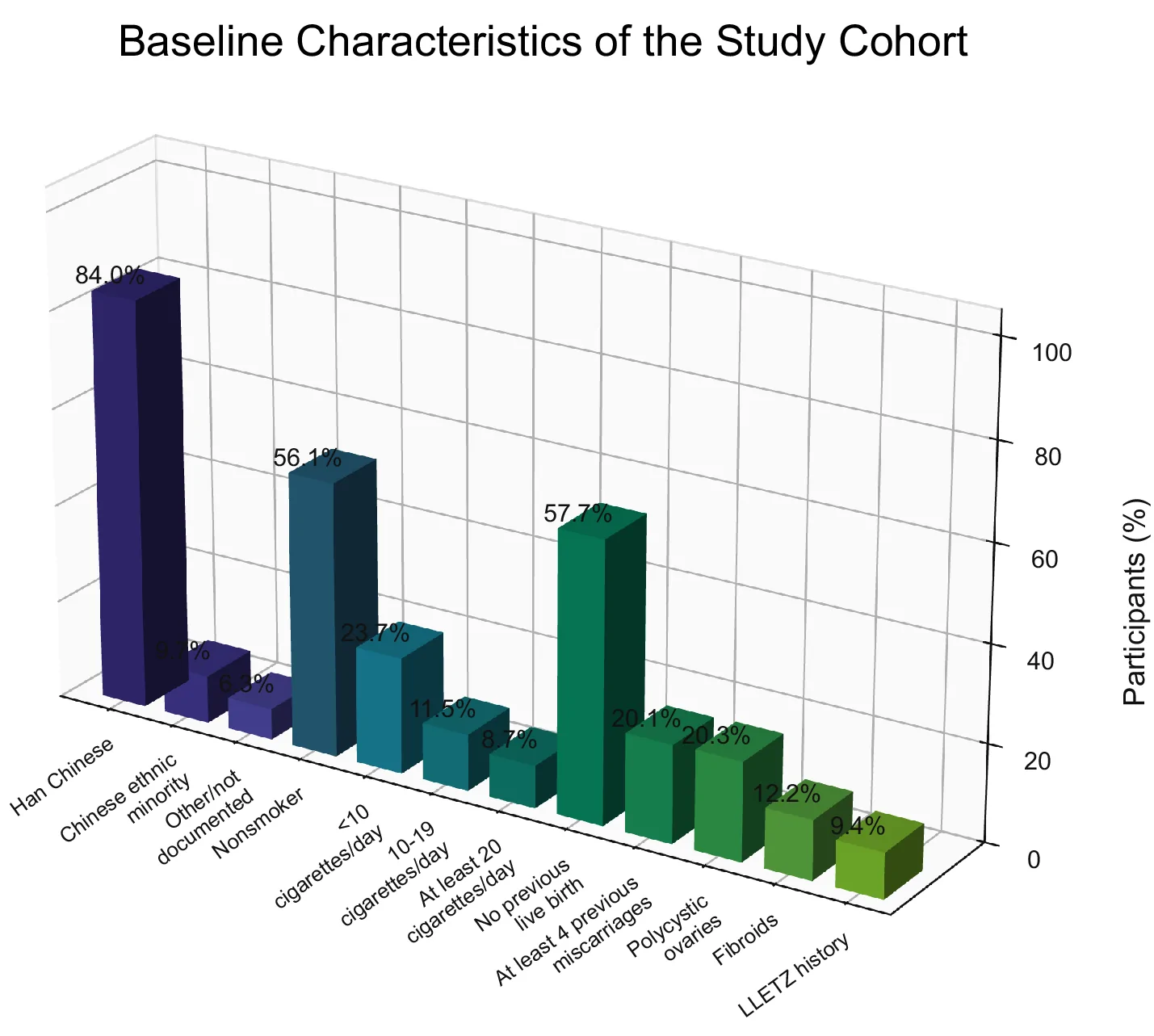
Baseline characteristics of the study cohort. Bars show percentages for ethnicity/nationality, smoking status, obstetric history, and gynecologic factors. The ethnicity/nationality categories are Han Chinese (84.0%), Chinese ethnic minority (9.7%), and other/not documented (6.3%). Percentages are based on available cases; denominators vary because of missing data.

**Table 1:** Baseline characteristics.

Variable		Descriptives
Maternal age (years), median (Q1, Q3)		32 (22, 35)
Maternal BMI (kg/m2), median (Q1, Q3)		22.9 (20, 25.1)
Maternal ethnicity/nationality, *n* (%)	Chinese ethnic minority	29 (9.7)
	Other/not documented	19 (6.3)
	Han Chinese	252 (84)
Mode of conception, *n* (%)	Spontaneous conception	300 (100.0)
Maternal smoking, *n* (%)	Nonsmoker	161 (56.1)
	<10 cigarettes/day	68 (23.7)
	10 to 19 cigarettes/day	33 (11.5)
	≥20 cigarettes/day	25 (8.7)
Alcohol use, *n* (%)	No current alcohol use	127 (46)
	≤3 units/day	91 (33)
	4–7 units/day	33 (12)
	>7 units/day	25 (9.1)
Previous live births, *n* (%)	No	169 (57.7)
	Yes	124 (42.3)
≥4 previous miscarriages, *n* (%)	No	235 (79.9)
	Yes	59 (20.1)
Previous pregnancy losses, median (Q1, Q3)		3 (2, 4)
Polycystic ovaries, *n* (%)	No	228 (79.7)
	Yes	58 (20.3)
Fibroids, *n* (%)	No	259 (87.8)
	Yes	36 (12.2)
Large-loop excision of cervical transf. Zone, *n* (%)	No	269 (90.6)
	Yes	28 (9.4)

Ethnicity/nationality reflects routine hospital registration categories after source data-dictionary review. Percentages are based on available cases; denominators vary because of missing data. Several category totals are less than *n* = 300.

### 3.2 Endometrial receptivity (before vs. after treatment)

Uterine Doppler indices significantly improved after therapy: the median pulsatility index decreased from 3.00 (2.49–3.60) to 2.30 (1.73–2.85), the resistive index from 0.75 (0.67–0.80) to 0.66 (0.59–0.72), and the systolic-to-diastolic ratio from 2.98 (2.78–3.67) to 2.10 (1.87–2.46); all *p* < 0.001 (Table 2).

**Table 2:** Assessment of endometrial receptivity.

Parameter	Before treatment	After treatment	*p*-value
Pulsatility index	3 (2.49, 3.6)	2.3 (1.73, 2.85)	<0.001
Resistive index	0.75 (0.67, 0.8)	0.66 (0.59, 0.72)	<0.001
Systolic-to-diastolic ratio	2.98 (2.78, 3.67)	2.1 (1.87, 2.46)	<0.001

Data is presented as median (Q1, Q3).

### 3.3 Primary outcome

Live birth at or after 20 weeks of gestation occurred in 211/300 women (70.3%); 89 pregnancies (29.7%) did not reach this endpoint. In the multivariable model adjusted for maternal age, a higher pretreatment systolic-to-diastolic ratio remained independently associated with lower odds of live birth (adjusted OR = 0.34, 95% CI 0.22–0.52, *p* < 0.001). Maternal age was also associated with lower live-birth odds when modeled as a continuous covariate (adjusted OR per year = 0.94, 95% CI 0.89–0.99, *p* = 0.031). In the prespecified sensitivity analysis, age >35 years showed a directionally lower live-birth odds (adjusted OR = 0.62, 95% CI 0.31–1.24, *p* = 0.176), while the association between pretreatment systolic-to-diastolic ratio and live birth remained materially unchanged (adjusted OR = 0.35, 95% CI 0.23–0.53, *p* < 0.001) (Table 3).

**Table 3:** Primary outcome.

Number	Yes	211	No	89
Predictor	B	S.E.	*p*-value	Adjusted odds ratio (95% CI)
Live birth after 20 weeks of gestation				
Pretreatment systolic-to-diastolic ratio	−1.08	0.22	<0.001	0.34 (0.22–0.52)
Maternal age (per year)	−0.06	0.03	0.031	0.94 (0.89–0.99)
Sensitivity model: maternal age >35 years	−0.48	0.35	0.176	0.62 (0.31–1.24)
Sensitivity model: pretreatment systolic-to-diastolic ratio	−1.05	0.22	<0.001	0.35 (0.23–0.53)

### 3.4 Secondary outcomes

Secondary pregnancy outcomes were as follows: clinical pregnancy at 6–7 weeks, defined as an intrauterine gestational sac with fetal cardiac activity, was observed in 99.3% of participants. All pregnancies analyzed for the primary and secondary pregnancy outcomes were spontaneous conceptions. Ectopic pregnancy was recorded separately in 5.3% and was not counted as intrauterine clinical pregnancy. Live birth at or after 24 weeks occurred in 69.3% of cases, live birth at 34 weeks or beyond occurred in 57.3%, and preterm birth occurred in 15.0%. Miscarriage and stillbirth were recorded in 29.7 and 7.0%, respectively (Figure 3). Among neonates, all infants with discharge data were discharged alive (210/210; 100%), median birth weight was 3,012 g (2491–3,446), and congenital anomalies were documented in 28 (12.6%) (Table 4). Safety assessments showed maternal adverse drug events in 48.5% of participants and serious adverse events in 42.7%; anti-drug antibody formation was detected in 20.2% (Figure 4). These safety classifications were retained from source charts and require careful interpretation because event severity may have been recorded differently across clinical departments.

**Table 4:** Secondary neonatal outcomes.

Secondary neonatal outcome		Descriptives
Infant birth weight (g), median (Q1, Q3)		3,012 (2,491, 3,446)
Infants discharged alive from hospital, *n* (%)	No	0
	Yes	210 (100)
Any congenital anomaly, *n* (%)	No	195 (87.4)
	Yes	28 (12.6)

**Figure 3:**
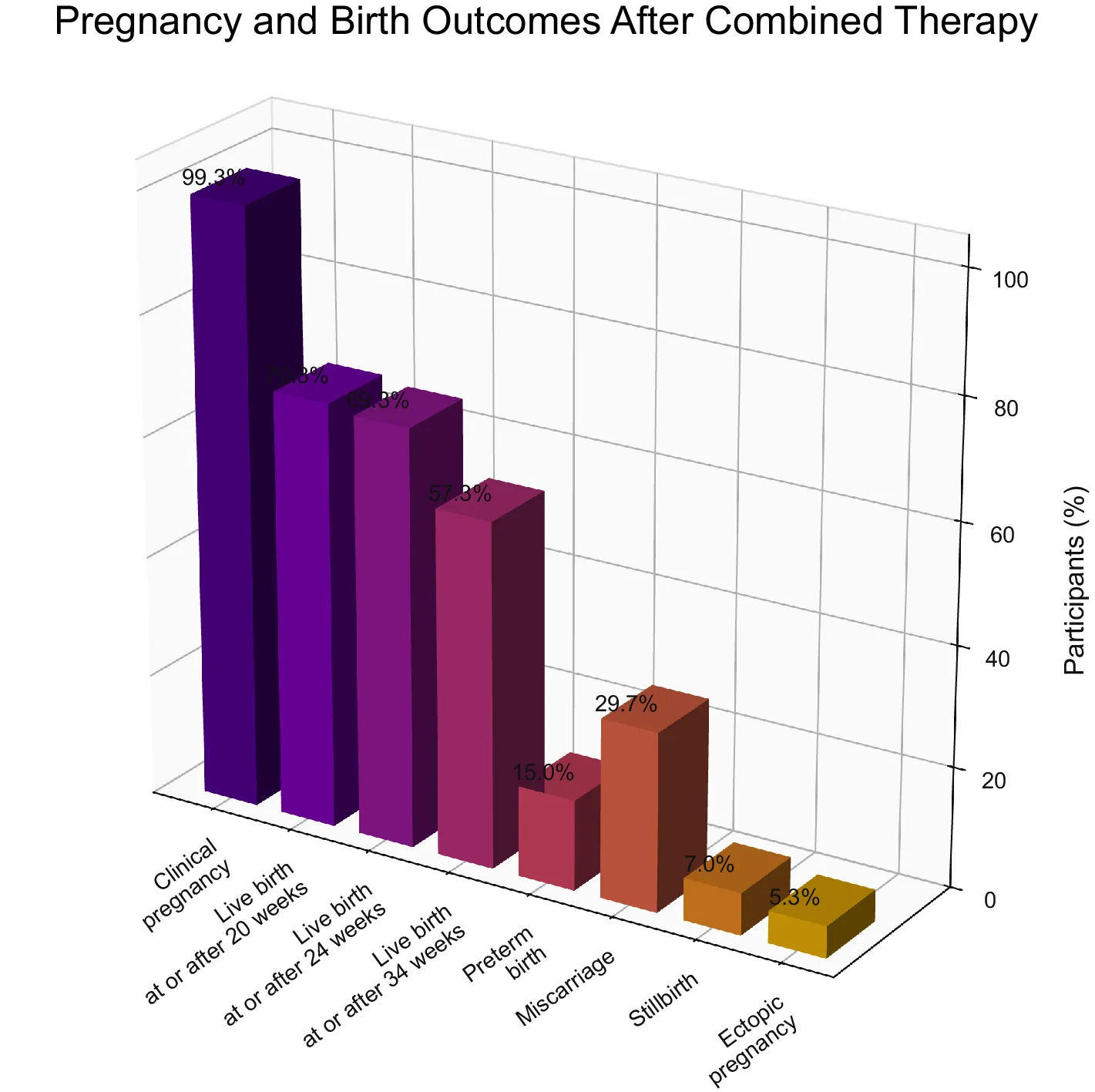
Secondary pregnancy outcomes.

**Figure 4:**
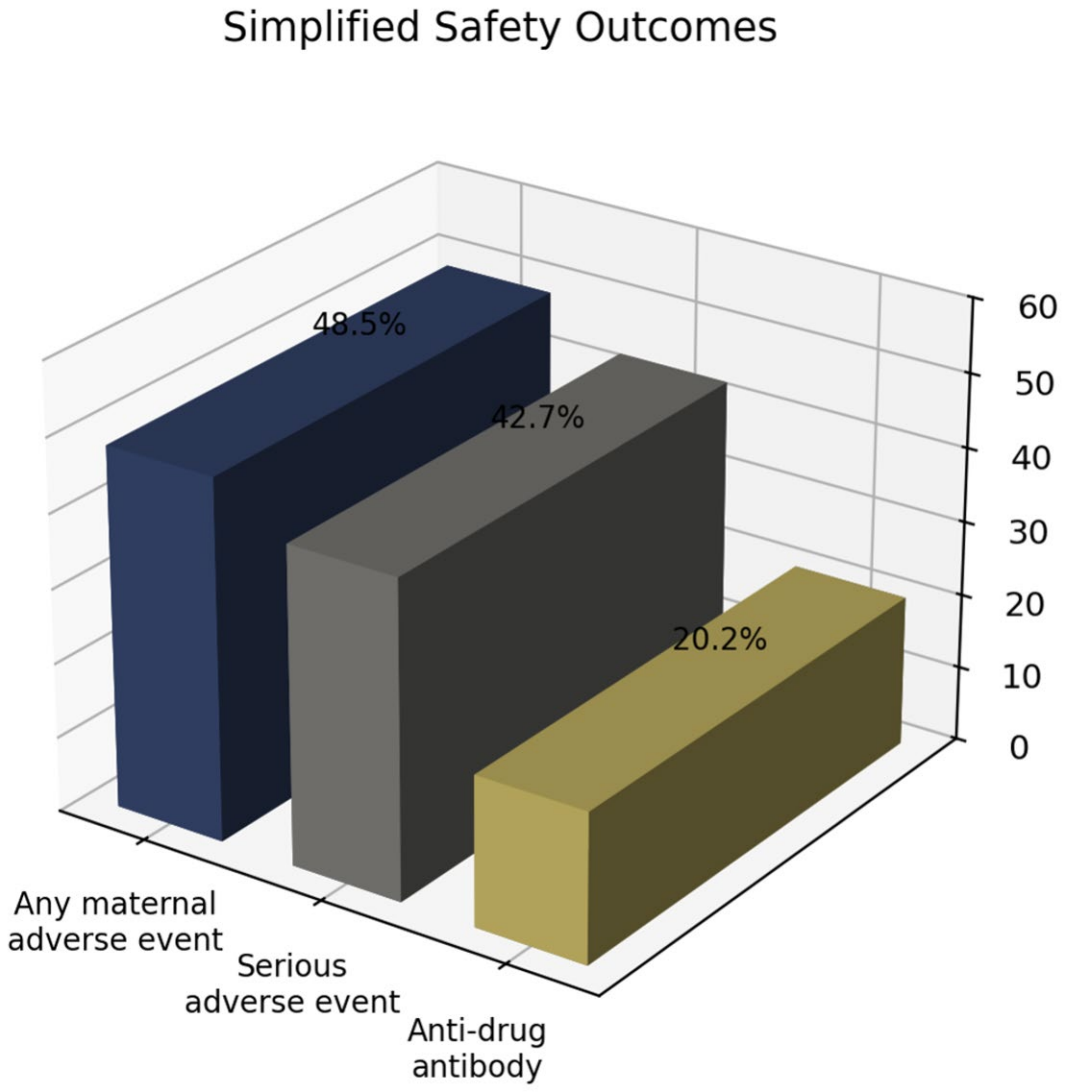
Safety outcomes.

## 4 Discussion

This retrospective study evaluated pregnancy outcomes after combined rhG-CSF and low-dose aspirin therapy in women diagnosed with unexplained RPL. The cohort achieved a 70.3% live-birth rate at or after 20 weeks and showed significant improvements in uterine artery Doppler parameters. These findings contribute to the evolving evidence regarding immune-modulatory and vascular interventions in RPL management, but the results should not be interpreted as proof of treatment efficacy because no untreated or aspirin-only control group was available.

The observed live-birth rate of 70.3% is clinically important for women with RPL and is broadly consistent with the retrospective study by Sun et al., in which combined rhG-CSF and aspirin was associated with a live-birth rate of 81.8% compared with 61.5% in controls (14). However, interpretation must be cautious. Retrospective cohorts receiving supportive care alone have reported high subsequent live-birth rates in women with unexplained recurrent first-trimester miscarriage, suggesting that some favorable outcomes may occur independently of pharmacologic intervention (18, 19). Trial evidence is also conflicting: Scarpellini and Sbracia reported a higher rate of healthy live birth after G-CSF than placebo (9), whereas Eapen et al. (10) found no improvement with rhG-CSF compared with placebo (59.2% vs. 64.9%). In addition, regulatory information for filgrastim products states approved indications related to neutropenia, stem-cell mobilization, and radiation exposure rather than RPL, and the 2022 ESHRE guideline does not recommend G-CSF for unexplained RPL because evidence of benefit is insufficient (11, 15). Published pregnancy data in severe chronic neutropenia registries have not shown major safety differences between G-CSF-exposed and unexposed pregnancies, but those populations differ substantially from women with unexplained RPL (16, 17).

Doppler indices of uterine artery after combination treatment significantly improved compared to baseline with reduction of pulsatility index from 3.00 to 2.30, resistive index from 0.75 to 0.66, and systolic/diastolic ratio from 2.98 to 2.10, indicating enhanced endometrial perfusion. Elevated uterine artery impedance has been consistently associated with RPL, and several studies have reported significantly higher pulsatility index, resistive index, and systolic-to-diastolic ratio values in patients with RPL than in controls (20, 21). Optimal endometrial receptivity appears to occur when the mean pulsatility index is between 2 and 3, whereas values exceeding 4 significantly decrease implantation and pregnancy rates (22). The median pulsatility index in our study was 2.30, falling within the safe range and suggesting favorable pregnancy outcomes. On conducting multivariate analysis, we observed that higher pre-treatment Systolic/Diastolic ratio correlated with reduced live birth odds, highlighting the prognostic significance of baseline uterine perfusion. This finding aligns with a previous study supporting a strong positive correlation between elevated uterine artery pulsatility index and recurrent miscarriage (*r* = 0.8, *p* < 0.001) (23).

A high clinical pregnancy rate of approximately 99% was observed at 6 weeks of gestation, with a miscarriage rate of 29.7%, suggesting successful implantation but a persistent risk of pregnancy loss in early gestation. These findings suggest that factors other than endometrial receptivity, such as the dormant embryo phenomenon, may contribute to pregnancy loss (24). The ectopic pregnancy rate of 5.3% exceeds the 1%–2% rate generally reported in natural conceptions and may reflect unmeasured tubal pathology or referral bias in this recurrent-pregnancy-loss cohort (25). Importantly, ART status was verified from source records, and no pregnancies achieved through IVF, ICSI, IUI, or other ART procedures were included in the primary analysis.

The preterm birth rate in our study was 15%, which falls within the global rate of preterm birth (26), suggesting no excess risk attributable to the combined therapy. The stillbirth rate of 7.0% appears elevated compared with high-income-country rates of 3-4 per 1,000, although it is comparable to rates in some low- and middle-income countries (27). The live birth rate after 24 weeks (69.3%) versus after 20 weeks (70.3%) indicates minimal losses between 20 and 24 weeks.

The median birth weight of 3,012 g was within the normal range. The congenital anomaly rate was 12.6%, which is relatively high; some reports indicate rates of approximately 5.5% (28). The higher rate in our cohort may be associated with multiple factors, such as the high rate of alcohol use during the study or potential treatment-related effects. Eapen et al. (10) reported congenital anomalies in 2.1% of the rhG-CSF group and 2.0% of the control group, a non-significant difference. Maternal adverse drug events and serious adverse events were recorded in 48.5% and 42.7% of participants, respectively; anti-drug antibodies were recorded in 20.2%.

### 4.1 Strengths and limitations

This study involved a relatively large sample of 300 participants, comprehensive outcome assessment, detailed Doppler evaluation, and multivariable modeling with prespecified maternal-age adjustment. We also verified mode of conception from source obstetric and fertility records and confirmed that all index pregnancies included in the primary analysis were spontaneous conceptions. Several limitations should be emphasized. First, the lack of an untreated, aspirin-only, or standard-care control group introduces substantial risk of selection bias, confounding by indication, and overestimation of treatment benefit. Second, maternal age, alcohol use, smoking, and Doppler measurements may be prone to residual confounding, misclassification, and inter-observer variability despite adjustment. Third, the demographic variable was derived from routine hospital registration data rather than a standardized research race/ethnicity questionnaire; therefore, we revised the wording to ethnicity/nationality and clarified the local classification system. Fourth, external validity may be limited because the cohort was recruited from two hospitals in Jiangxi, China and was predominantly Han Chinese. Finally, the relatively high rates of recorded congenital anomalies and serious adverse events require verification in prospective studies with standardized safety adjudication.

### 4.2 Clinical implications and recommendations

The findings suggest that combined rhG-CSF and aspirin may warrant further investigation in selected RPL patients, particularly those with impaired uterine perfusion on ultrasound assessment. However, because rhG-CSF use for unexplained RPL is off-label and not recommended routinely by current ESHRE guidance, clinical application should be individualized and accompanied by counseling regarding uncertain efficacy, potential adverse events, and the need for close monitoring (11, 15). Cost-effectiveness is also relevant because rhG-CSF is expensive and requires repeated administration. Future randomized or well-controlled comparative studies should evaluate rhG-CSF plus aspirin against aspirin alone and standard supportive care, include prespecified maternal-age adjustment, define clinical pregnancy and live birth consistently, stratify spontaneous and ART pregnancies when both are present, and include standardized maternal and neonatal safety adjudication.

## 5 Conclusion

In this retrospective cohort of women with unexplained RPL, combined rhG-CSF and low-dose aspirin was associated with improved uterine artery Doppler parameters and a 70.3% live-birth rate at or after 20 weeks. The association between pretreatment systolic-to-diastolic ratio and live birth persisted after maternal-age adjustment, and all pregnancies included in the primary analysis were verified as spontaneous conceptions. The findings are hypothesis-generating rather than definitive because there was no control group and current guideline evidence does not support routine rhG-CSF use for unexplained RPL. Prospective controlled studies are required to confirm efficacy, validate the age-adjusted associations, standardize definitions of clinical pregnancy and live birth, and evaluate maternal and neonatal safety, particularly the recorded congenital anomaly rate.

## Data Availability

The raw data supporting the conclusions of this article will be made available by the authors, without undue reservation.

## References

[1] Eleje GU Ugwu EO Igbodike EP Malachy DE Nwankwo EU Ugboaja JO . Prevalence and associated factors of recurrent pregnancy loss in Nigeria according to different national and international criteria (ASRM/ESHRE vs. WHO/RCOG). Front Reprod Health. (2023) 5:1049711. doi: 10.3389/frph.2023.1049711, 36895657 PMC9989171

[2] Laijawala RA. Recurrent pregnancy loss: immunological aetiologies and associations with mental health. Brain Behav Immun Health. (2024) 41:100868. doi: 10.1016/j.bbih.2024.100868, 39391794 PMC11466561

[3] Youssef A Lashley L Dieben S Verburg H Van Der Hoorn M-L. Defining recurrent pregnancy loss: associated factors and prognosis in couples with two versus three or more pregnancy losses. Reprod Biomed Online. (2020) 41:679–85. doi: 10.1016/j.rbmo.2020.05.016 32811769

[4] Boedeker D Hunkler K Mahdy H. "Recurrent pregnancy loss". In: StatPearls. Treasure Island, FL: StatPearls Publishing (2025) 32119347

[5] Marozio L Nuzzo AM Gullo E Moretti L Canuto EM Tancredi A . Immune checkpoints in recurrent pregnancy loss: new insights into a detrimental and elusive disorder. IJMS. (2023) 24:13071. doi: 10.3390/ijms241713071, 37685876 PMC10488095

[6] Bagkou Dimakou D Lissauer D Tamblyn J Coomarasamy A Richter A. Understanding human immunity in idiopathic recurrent pregnancy loss. Eur J Obstet Gynecol Reprod Biol. (2022) 270:17–29. doi: 10.1016/j.ejogrb.2021.12.024, 35007974

[7] Fu L Xu Y Yan J Zhang X Li D Zheng L. Efficacy of granulocyte colony-stimulating factor for infertility undergoing IVF: a systematic review and meta-analysis. Reprod Biol Endocrinol. (2023) 21:34. doi: 10.1186/s12958-023-01063-z, 37013570 PMC10069139

[8] Mu F Cai Z Du L Zeng X Zhou Q Chen Y . Efficacy and safety of recombinant human granulocyte colony-stimulating factor in patients with unexplained recurrent pregnancy loss: a protocol for a multicenter, randomized, double-blind, controlled trial. Trials. (2025) 26:245. doi: 10.1186/s13063-025-08951-2, 40660339 PMC12257775

[9] Scarpellini F Sbracia M. Use of granulocyte colony-stimulating factor for the treatment of unexplained recurrent miscarriage: a randomised controlled trial. Hum Reprod. (2009) 24:2703–8. doi: 10.1093/humrep/dep240, 19617208

[10] Eapen A Joing M Kwon P Tong J Maneta E De Santo C . Recombinant human granulocyte–colony stimulating factor in women with unexplained recurrent pregnancy losses: a randomized clinical trial. Hum Reprod. (2019) 34:424–32. doi: 10.1093/humrep/dey393, 30776296 PMC6389865

[11] Bender Atik R Christiansen OB Elson J Kolte AM Lewis S Middeldorp S . ESHRE guideline: recurrent pregnancy loss: an update in 2022. Hum Reprod Open. (2022) 2023:hoad002. doi: 10.1093/hropen/hoad002, 36873081 PMC9982362

[12] Mu F Huang J Zeng X Liu L Wang F. Efficacy and safety of recombinant human granulocyte colony-stimulating factor in patients with unexplained recurrent spontaneous abortion: a systematic review and meta-analysis. J Reprod Immunol. (2023) 156:103830. doi: 10.1016/j.jri.2023.103830, 36821985

[13] Toth B Vomstein K Togawa R Böttcher B Hudalla H Strowitzki T . The impact of previous live births on peripheral and uterine natural killer cells in patients with recurrent miscarriage. Reprod Biol Endocrinol. (2019) 17:72. doi: 10.1186/s12958-019-0514-7, 31472670 PMC6717647

[14] Sun S Chen J Gao M Li G Xu C Sun M . Impact of recombinant human granulocyte colony-stimulating factor combined with aspirin on clinical pregnancy outcomes and endometrial receptivity in patients with recurrent abortion: a retrospective study. Altern Ther Health Med. (2024) 30:205–9. 37773678

[15] United States Food and Drug Administration. NEUPOGEN (Filgrastim) Prescribing Information. Silver Spring, MD: FDA (2025).

[16] Boxer LA Bolyard AA Kelley ML Marrero TM Phan L Bond JM . Use of granulocyte colony-stimulating factor during pregnancy in women with chronic neutropenia. Obstet Gynecol. (2015) 125:197–203. doi: 10.1097/AOG.0000000000000602, 25560125 PMC4286310

[17] Zeidler C Grote UAH Nickel A Brand B Carlsson G Cortesão E . Outcome and management of pregnancies in severe chronic neutropenia patients by the European branch of the severe chronic neutropenia international registry. Haematologica. (2014) 99:1395–402. doi: 10.3324/haematol.2013.099101, 24997149 PMC4116840

[18] Costa OLN Santos EMF Jesus VS de Netto EM (2015) Reproductive outcome in pregnant women with recurrent pregnancy loss Rev Bras Ginecol Obstet 37:578–584 doi: 10.1590/SO100-720320150005445 26650322

[19] Linehan LA San Lazaro Campillo I Hennessy M Flannery C O’Donoghue K. Reproductive outcomes following recurrent first-trimester miscarriage: a retrospective cohort study. Hum Reprod Open. (2022) 2022:hoac045. doi: 10.1590/S0100-720320150005445, 36339248 PMC9632453

[20] Zhong Y Wang N Lu S Lu Y Pan X Zhou Y. Doppler evaluation of uterine blood flow in patients with unexplained recurrent pregnancy loss. IJWH. (2024) 16:1803–14. doi: 10.2147/IJWH.S477828, 39493660 PMC11531733

[21] Yang W Wu Z Yu M Peng X Lu W Feng W . Characteristics of midluteal phase uterine artery hemodynamics in patients with recurrent pregnancy loss. J Obstet Gynaecol. (2019) 45:1230–5. doi: 10.1111/jog.13944, 30977230

[22] Aslam T Khadija S Hafeez A Rauf M Saleem Z Hameed S. Transvaginal sonographic evaluation of Doppler indices of ovarian artery and uterine artery in infertile females (2021) 19:15–9.

[23] Hamed S Shedid A Abdel-Halim G Zainel-Din A. Study of the changes of pulsatility index (PI) in uterine artery in patients with recurrent pregnancy loss. Evid Based Women’s Health J. (2024) 14:55–60. doi: 10.21608/ebwhj.2023.245173.1269

[24] Bulletti FM Sciorio R Conforti A De Luca R Bulletti C Palagiano A . Causes of embryo implantation failure: a systematic review and metaanalysis of procedures to increase embryo implantation potential. Front Endocrinol. (2025) 15:1429193. doi: 10.3389/fendo.2024.1429193, 40028443 PMC11867936

[25] Panelli DM Phillips CH Brady PC. Incidence, diagnosis and management of tubal and nontubal ectopic pregnancies: a review. Fertil Res Pract. (2015) 1:15. doi: 10.1186/s40738-015-0008-z, 28620520 PMC5424401

[26] Ohuma EO Moller A-B Bradley E Chakwera S Hussain-Alkhateeb L Lewin A . National, regional, and global estimates of preterm birth in 2020, with trends from 2010: a systematic analysis. Lancet. (2023) 402:1261–71. doi: 10.1016/S0140-6736(23)00878-4, 37805217

[27] Hug L You D Blencowe H Mishra A Wang Z Fix MJ . Global, regional, and national estimates and trends in stillbirths from 2000 to 2019: a systematic assessment. Lancet. (2021) 398:772–85. doi: 10.1016/S0140-6736(21)01112-0, 34454675 PMC8417352

[28] Timilsina D Gupta S. Prevalence of congenital anomalies among pregnant women at a tertiary care center: a cross-sectional study. Cureus. (2025) 17:e79444. doi: 10.7759/cureus.79444, 40130102 PMC11931595

